# Integrin beta1 mediates the effect of telocytes on mesenchymal stem cell proliferation and migration in the treatment of acute lung injury

**DOI:** 10.1111/jcmm.17976

**Published:** 2023-10-19

**Authors:** Ruixue Qi, Jiayun Hou, Ying Yang, Zhicheng Yang, Lihong Wu, Tiankui Qiao, Xiangdong Wang, Dongli Song

**Affiliations:** ^1^ Jinshan Hospital Center for Tumor Diagnosis & Therapy, Jinshan Hospital Fudan University Shanghai Medical School Shanghai China; ^2^ Zhongshan Hospital, Department of Pulmonary and Critical Care Medicine, Institute of Clinical Science Fudan University Shanghai Medical School Shanghai China; ^3^ Shanghai Engineering Research Center of AI Technology for Cardiopulmonary Diseases Shanghai China; ^4^ Shanghai Institute of Clinical Bioinformatics Shanghai Key Laboratory of Lung Inflammation and Injury Shanghai China; ^5^ Department of Pulmonary Medicine Shanghai Xuhui Central Hospital, Fudan University Shanghai China

**Keywords:** acute lung injury, integrin beta1, lung, MSCs, PI3K, telocytes

## Abstract

Co‐transplantation of mesenchymal stem cells (MSCs) with telocytes (TCs) was found to have therapeutic effects, although the mechanism of intercellular communication is still unknown. Our current studies aim at exploring the potential molecular mechanisms of TCs interaction and communication with MSCs with a focus on integrin beta1 (ITGB1) in TCs. We found that the co‐culture of MSCs with ITGB1‐deleted TCs (TC^*ITGB1‐ko*^) changed the proliferation, differentiation and growth dynamics ability of MSC in responses to LPS or PI3K inhibitor. Changes of MSC proliferation and apoptosis were accompanied with the dysregulation of cytokine mRNA expression in MSCs co‐cultured with TC^*ITGB1‐ko*^ during the exposure of PI3Kα/δ/β inhibitor, of which IL‐1β, IL‐6 and TNF‐α increased, while IFN‐γ, IL‐4 and IL‐10 decreased. The responses of PI3K p85, PI3K p110 and pAKT of MSCs co‐cultured with TC^*ITGB1‐ko*^ to LPS or PI3K inhibitor were opposite to those with ITGB1‐presented TCs. The intraperitoneal injection of TC^*ITGB1‐ko*^, TC^*vector*^ or MSCs alone, as well as the combination of MSCs with TC^*ITGB1‐ko*^ or TC^*vector*^ exhibited therapeutic effects on LPS‐induced acute lung injury. Thus, our data indicate that telocyte ITGB1 contributes to the interaction and intercellular communication between MSCs and TCs, responsible for influencing other cell phenomes and functions.

## 1 INTRODUCTION

There is growing evidence that telocytes (TCs), as a new type of interstitial cells, take a significant part in supporting lung tissue constructions, connecting different cells and coordinating cell–cell communications, since TCs were found in human trachea and lungs.^19^ Telocytes exist along with basement of airway and alveolar epithelial cells, between smooth muscle cells, epithelial cells and fibroblasts, and/or in alveolar space, blood vessels and bronchiole. On the basis of telocyte biological characteristics and morphological features, TCs were considered to take an important part in the pathogenesis of lung diseases and to act as a supportive candidate and alternative of therapies for lung injury.^6^, ^13^ In addition, telopodes, as the extensive branching from cytoplasm with podoms and podomeres, form a three‐dimensional network structure connecting with other types of cells, such as stem cells and immune cells. It is possible that telopodes of implanted TCs form networks with endothelial cells like the interaction with neuronal circuits,^10^ or produce growth factors through activating PI3K isoform signaling pathways like PI3Kα/δ/β, PI3K p110δ, PI3K/mTOR or pan‐PI3K.^15^

Our previous studies evidenced that combined transplantation of mesenchymal stem cells (MSCs) and TCs could prevent and treat experimental lung tissue inflammation, oedema and injury.^19^ Intraperitoneal injection of TCs increased the proliferation and motility of MSCs—from intraperitoneal cavity or intratracheal space into lung injured area through the activation of osteopontin‐dominant networks and interaction between epidermal growth factors and corresponding receptors to support implanted MSCs. Intratracheal administration with TCs was found to reduce lung inflammation and tissue injury and improve the lung function in mechanical ventilation‐induced acute lung injury, through the maintenance of microvascular endothelial barrier function and production of vascular endothelial growth factor (VEGF) from TCs.^7^ The inflammation could alter the expression of microRNA and mRNA, transcriptomic profiles and responses of lung TCs per se to stimulators or inhibitors (e.g. miR‐21a‐3p inhibition), leading to lung tissue repair and angiogenesis in LPS‐induced acute lung injury, rather than conditional culture medium from TCs with miR‐21a‐3p inhibitor.^20^ The present studies aim at investigating potential molecular mechanisms by which TCs interact and communicate with MSCs with a focus on integrin beta1 (ITGB1) in TCs, by measuring MSCs proliferation dynamics, apoptosis and differentiation after co‐culture with TCs or ITGB1‐deleted TCs in responses to LPS or PI3K inhibitor. We also monitored the roles of ITGB1‐deleted TCs in the cytokine production, mitochondrial function, and intracellular signaling pathways of MSCs, as well as therapeutic effects in experimental acute lung injury after co‐transplantation of MSCs with TCs or ITGB1‐delated TCs.

## 2 MATERIALS AND METHODS

### 2.1 Culture of TCs and MSCs

The mouse lung TCs were isolated and constructed (TC^*SV40*^) as described before.^18^ The TCs were cultivated in DMEM (Dulbecco's modified Eagle's medium)/F12 (Gibco; Thermo Fisher Scientific, Inc.) in the incubator with 5% CO_2_ at 37°C. The DMEM/F12 medium was added with 10% fetal bovine serum purchased from Gibco, streptomycin at 0.1 mg/mL (Sigma‐Aldrich) and penicillin at 100 UI/mL. The human MSCs were purchased from BioCell Company. The immunophenotype and pluripotency of stem cells were identified by the suppliers. MSCs were cultured in the specific stem cell culture medium with 5% fetal bovine serum purchased from Gibco, growth factors, penicillin at 100 U/mL, and streptomycin at 0.1 mg/mL (Sigma‐Aldrich). When the cells reach the density of 60%–70%, they were subcultured, after washing with PBS, then digested with trypsin (0.25%), collected in the test tubes and centrifuged (400 × ***g***) for 5 min, then inoculated in culture bottles. The cells we used for experiments were within Generations 3–10.

### 2.2 Immunofluorescent staining

As mentioned earlier, we performed double immunofluorescence staining for CD34, vimentin and PDGFRα. In short, TCs were cultured on the bottom of 20 mm diameter cell culture dishes purchased from NEST and fixed in the 4% paraformaldehyde with triton‐X‐100 (0.05%) for about 20 min. After the cells were washed with PBS thrice, they were blocked in 5% BSA (bovine serum albumin) for 1 h and cultured with mouse anti‐CD34 antibody (1:100 dilution; 5.5 μg/mL; Abcam), rabbit anti‐PDGFRα (1:200 dilution; 5 μg/mL; Abcam) and goat anti‐vimentin antibody (1:200 dilution; 1.2 μg/mL; Abcam) diluted with 1% BSA in PBS at 4°C overnight. After washing with PBS for three times, the cells were incubated with PE bound anti mouse secondary antibody. FITC bound anti‐goat secondary antibody (1:200 dilution; 20 μg/mL; Jackson ImmunoResearch) and APC bound anti‐rabbit secondary antibody (1:200 dilution; 10 μg/mL; Jackson ImmunoResearch). The nuclei were labelled with DAPI staining, on the basis of the manufacture's instruction (KeyGEN BioTECH).

### 2.3 ITGB1 lentivirus construction and infection

We constructed lentivirus particles containing ITGB1 gene. Mus ITGB1 open reading frame (ORF) was cloned from 293 T reverse transcription cDNA with forward primer 5’‐CCACAGAAGTTTACATTAA‐3′ and reverse primer 5′‐TTAATGTAAAGTTCTGTGG‐3′. XhoI and BamHI restriction sites were put in the forward and reverse primers, respectively, and used to clone ITGB1 ORF into MV‐MCS‐PGK‐Blasticidin (PHY‐009) vector to generate plasmid. The oligo including negative Control forward: 5′‐TTCTCCGAACGTGTCACGT‐3′ and reverse: 5′‐ACGAGACACGTTCGGAGAA‐3′ were constructed to generate plasmid as reported previously.^14^ TC^*SV40*^ cells were inoculated with 10^5^ cells/well density, cultured at 37°C for 24 h, then replaced with fresh medium. The recombinant lentivirus vector was added with multiplicity of infection (MOI = 10) after 24 h of culture and puromycin 3 μg/mL was added on the third day of infection. ITGB1‐negative cells were selected and colonized as TC^*ITGB1‐ko*^ cells for follow‐up experiments.

### 2.4 Roles of phosphoinositide 3‐kinase (PI3K) in TC‐MSC interactions

TC^*SV40*^ cells and MSCs in logarithmic growth period were co‐cultured in Transwell device (0.4 μm aperture; Corning costar) (*n* = 6/group) to prevent physical contact between TC^*SV40*^ cells and MSCs, where TC^*SV40*^ cells were loaded in the upper chamber, and MSCs at 10^5^ cells/well in the lower. MSC behaviour was influenced by TC conditioned medium. MSCs alone were stimulated with vehicle as the negative control or lipopolysaccharide (LPS) at 1 μg/mL for 24 and 48 h, respectively. TC^*SV40*^ cells and MSCs (TC/MSC) or TC^*ITGB1‐ko*^ cells and MSCs (TC^*ITGB1‐ko*^ /MSC) were co‐cultured in the Transwell overnight and then stimulated with vehicle or LPS, respectively. To evaluate the role of PI3K catalytic isoform proteins, MSCs alone, TC/MSC or TC^*ITGB1‐ko*^/MSC were both stimulated with LY294002 (a kind of PI3Kα/δ/β inhibitor from Selleck Chem) at 0.5 μM for 24 h and followed by treated with LPS at 1 μg/mL.

### 2.5 Protein measurement

Cells were lysed with radio immunoprecipitation assay (RIPA) and phenylmethanesulfonyl fluoride (PMS) at 4°C and centrifugated at 14000 ***g*** for 10 min, of which the supernatant solution was harvested and protein was extracted to determine the amount of total protein using BCA Protein Assay Kit purchased from KeyGEN BioTECH Co., Ltd). SDS‐PAGE separation gel was prepared according to the instruction of Gel preparation kit (Kaiji Biotechnology Co.). Then, 20 μg proteins were taken and separated by the 10% SDS‐PAGE gradients purchased from Life technologies, then transferred to the polyvinylidene fluoride (PVDF) membranes from Millipore. After being blocked with TBS‐T solution (Tween 20 in Tris‐buffered saline with 5% milk) for 1 h, the gel was added with anti‐ITGB1 (1:500) and anti‐GAPDH (1:1000) primary antibodies at 4°C through the night, washed with TBS‐T buffer for 5 min thrice, incubated with secondary antibodies at room temperature for an hour, then monitored with Electrochemiluminescence (ECL) light‐emitting reagent dropwise. The amount of protein bands was analysed and quantified with one image analysis software as the optical density value.

### 2.6 Protein level of Bcl‐xl, P65, PI3K, AKT in MSC was measured

The experimental procedures have been described previously. After MSC lysis, total proteins were gathered and then run on 10% SDS‐PAGE gradient gels or 12% gradient gels. The membranes were incubated overnight at 4°C with the following primary antibodies: anti‐Bcl‐xl (1:20,000; 0.934 μg/mL), anti‐P‐P65(0.1 μg/mL), anti‐P65(1:2000; 0.5 μg/mL), anti‐PI3K(p110) (1:1000; 1.5 μg/mL), anti‐PI3K (85) (1:1000; 0.95 μg/mL), anti‐AKT (1:10,000; 0.18 μg/mL) and anti‐P‐AKT(1:5000). Then, membranes were washed and incubated with a secondary antibody (goat anti‐rabbit) (1:20,000; 0.1 μg/mL). The antibodies mentioned were all from Abcam. An enhanced chemiluminescence system (ECL, Pierce) is used to display the bands.

### 2.7 Identification of MSC surface markers

The surface markers of CD29, CD44 and CD133 on MSCs were detected using flow cytometry. MSCs were gathered and then washed with PBS twice after centrifugated for 5 min at 1000 rpm, then re‐suspended with 90 μL PBS. According to the antibody instructions: CD29‐PE (Thermo Fisher Scientific), FL2 channel was used for detection; CD44‐ FITC (BioLegend), FL1 channel was used for detection; CD133‐ APC (BioLegend), received as FL4 channel, an appropriate amount of antibody was added and incubated in the dark at for 30 min. After adding 400 μL PBS, the cell phenotypes of CD29, CD44 and CD133 were detected by Becton‐Dickinson FACS Calibur (BD Biosciences).

### 2.8 Measurement of MSC interval proliferation

After co‐cultured TC^*SV40*^ cells or TC^*ITGB1‐ko*^ cells with MSCs in Transwell device (0.4 μm aperture) and treated with LPS for 24 h or 48 h. Telocytes and MSCs were digested and cultured in the 96‐well co culture plates at 5 × 10^3^/well (*n* = 6/group). Before the end of culture, 10 μL CCK8 reagents from Dojindo Molecular Technologies, Inc. were put into each well and then incubated for an additional 1 h. The absorbance value of the wave length was measured by enzyme scale at 450 nm. Then cell proliferation level was expressed by absorbance value.

### 2.9 Dynamic monitoring of MSC proliferation and migration

The bio‐behaviours of MSCs, including cell movement, cell morphology, total or dead cell numbers, were analysed by a Cell‐IQ system produced from Chip‐Man Technologies. The cells were photographed every 2 h for 50 h. The dynamics of MSC proliferation rate analysis was conducted using the imaging software (Biophotonics Facility, McMaster, Ontario, Canada). The manual tracking plug‐in was developed by France Cordelie's Institute. Using image analysis software, six fields were randomly selected from each hole for continuous photo taking and real‐time monitoring.

### 2.10 Detection of MSC proliferation by EdU‐absorption

After the cells were treated, all kinds of reagents were added according to the instructions of the kFluor594 Click‐iT EdU kit (Jiangsu KeyGEN BioTECH Co., Ltd.). Cells were pretreated with 10 μM of EdU for 2 h. Then cells were fixed in 4% paraformaldehyde and permeabilized by 0.5% triton X‐100 and 0.1% SDS. Hoechst33342 (Jiangsu KeyGEN BioTECH Co., Ltd.) were added for nucleus staining. Fluorescent images were captured and analysed using Operetta CLS™ high‐content screening (HCS, PerkinElmer) equipped with Opera QEHS Camera System (PerkinElmer) with Harmony software. Three independent experiments were performed.

### 2.11 Detection of MSC apoptosis and cell‐cycle

MSCs were digested by trypsin and centrifuged at 400 × ***g*** for 5 min, washed twice at 4°C, and then suspended with 300 μL binding buffer. Five microliters of annexin V‐FITC was added for the measurement of cell apoptosis and incubated at room temperature for 15 min in the dark. Before the measurement, the well was added with propidium iodide (PI) at 5 μL for 5 min and binding buffer at 200 μL. Flow cytometry analysed the results within an hour. All experiments were repeated three times. Cell cycle phases were tested by measuring the DNA fragment staining with propidium iodide (PI) purchased from Sigma‐Aldrich Co. According to the manufacturer's instruction, MSCs were digested with trypsin (0.25% concentration without EDTA) and washed with PBS twice (1000 rpm centrifuged for 5 min), then gathered for 5 min × 10^5^ cells; 70% ethanol was used to fix the prepared single cell suspension for at least 2 h (or overnight), then stored at 4°C before staining; plus 100 μL RNase A, 37°C water bath for 30 min; add another 400 μL PI dyeing and mixing, 4°C away from light for 30 min; test and record the red fluorescence with 488 nm of excitation wavelength on the machine.

### 2.12 Detection of mitochondrial membrane potential

The cells were digested with trypsin (0.25% concentration without EDTA) and washed with PBS once at 2000 rpm for 5 min, and the cells were collected and adjusted to 1 × 10^6^/mL; 100 μL 10 × incubation buffer was diluted to 1 × incubation buffer, mixed and then preheated to 37°C. Each 500 μL 1 × incubation buffer was mixed with 1 μL JC‐1 vortex to form JC‐1 working solution. The cells were evenly suspended in 500 μL JC‐1 working solution and cultured in the incubator conditioned with 37°C, 5% CO_2_ incubator for about 20 min. The collected cells were centrifuged (1000 rpm 5 min at room temperature)and washed with 1 × incubation buffer twice. Then the collected cells were resuspended with 500 μL 1 × incubation buffer and then tested by flow cytometry (Becton‐Dickinson FACS Calibur).

### 2.13 Determination of oxidative stress

After the cells were treated, all kinds of reagents were added according to the instructions of the kits. The content of malondialdehyde (MDA, Jiangsu KeyGEN BioTECH), superoxide dismutase (SOD, Jiangsu KeyGEN BioTECH) and glutathione (GSH, Jiangsu KeyGEN BioTECH) were detected. The enzyme reader was used to read at 450 nm. The inhibition rate of SOD and MDA was calculated according to the formula, respectively. The content of GSH was calculated according to the instructions. The content of T‐GDH and GSSG was calculated. The content of reduced glutathione (GSH) = the content of T‐GSH−2 × the content of GSSG.

### 2.14 Animal models

The study protocol was approved by Institutional Animal Care and Use Committee of Shanghai Laboratory Animal Center (2020072002). The mice, 8‐week‐old male C57BL/6, 22 to 25 g, were randomly divided into eight groups: vehicle, ALI, ALI with TCs^*vector*^ treatment, ALI with TCs^*ITGB1‐ko*^ treatment, ALI with L929 (fibroblast cell line) treatment, ALI with TCs^*vector*^ and MSC treatment, ALI with TCs ^*ITGB1‐ko*^ and MSC treatment, and ALI with MSC treatment. 10^6^ cells per mice of TCs, MSCs, L929 or TCs with MSCs were pretreated intraperitoneally under 60 mg/kg sodium pentobarbital anaesthesia purchased from Sinopharm Chemical Reagent Co (Shanghai, China). Twenty‐four hours later, mice were intratracheally instilled with PBS (phosphate‐buffered saline) as vehicle group or LPS from Sigma (Germany) at 5 mg/kg, via tracheal intubation after anaesthesia. After 24 h, the animals were perfused with 0.5 mL PBS, then killed and the lungs were gathered.

### 2.15 Haematoxylin and eosin staining and transmission electron microscopy

Twenty‐four hours after ALI induction, the lungs of mice were gathered for haematoxylin eosin staining. In brief, after endotracheal intubation, the lung tissues were fixed with neutral formaldehyde under 20 cm H_2_O pressure to maintain the alveolar structure. Lung tissue was fixed with formaldehyde and embedded in paraffin, 5–8 μm sections, Haematoxylin and eosin staining and then observed with the light microscope. According to the scoring criteria in Table S2, acute lung inflammation and injury were scored by histological analysis.

### 2.16 Transmission electron microscopy

As mentioned earlier, the ultrastructure of the cells was observed under transmission electron microscope (TEM).^17^ In brief, the lungs from vehicle, ALI, ALI with TCs^*vector*^ treatment, ALI with TCs^*ITGB1‐ko*^ treatment, ALI with L929 treatment, ALI with TCs^*vector*^ and MSC treatment, ALI with TCs ^*ITGB1‐ko*^ and MSC treatment, and MSCs with ALI treatment were gathered, and fixed for 4 h in 4% glutaraldehyde at pH 7.3, 4°C. Slides were washed with 0.1 M cacodylate buffer afterwards and post‐fixed in 0.1 M cacodylate buffer at pH 7.3, 4°C with 1% osmium tetroxide. After fixation, the slides were dehydrated in a graded series of ethanol, soaked through the night in a Epon 812 resin and mixture, and then embedded in Epon 812. Ultrathin sections of 70 nm were sliced through the Leica LKB‐II, produced from Nußloch (Germany), then gathered on the copper grids coated with Formvar and dyed with lead citrate and uranyl acetate, then observed by electron microscope produced from JEOL JEM‐1230 at an accelerating voltage of 80 kV.

### 2.17 Assay of cytokine genes

Protein levels and gene expression of cytokines were measured after cell culture, including interleukin IL‐4, IL‐1β, IL‐10, IFN (interferon) ‐γ and tumour necrosis factor (TNF)‐α. Total RNA of MSCs was isolated and reverse‐transcribed into single‐stranded cDNA with the 1st Strand cDNA Synthesis Kit for RT‐qPCR (reverse transcription quantitative PCR) (AMV, Roche Molecular Systems, Inc.) according to the recommendations. cDNA was synthesized from 1 μg of total RNA with Prime Script® RT reagent Kit purchased from Takara Bio Inc. RT‐qPCR was carried out with specific primers for the target genes, of which the sequences were listed in Table S1, and carried out on ABI PRISM 7300 Real‐time PCR System from Applied Biosystems with SYBR Premix Ex TaqTM from TaKaRa. The relative gene expression was quantified and all data were standardized on the basis of the relative expression level to analyse the folding differences.

### 2.18 Enzyme linked immunosorbent assay (ELISA)

TC^SV40^ cells and MSCs in logarithmic growth period were co‐cultured in Transwell device, TC^SV40^ cells were loaded in the upper chamber, and MSCs in the lower at 10^5^ cells/well. The cells supernatant were harvested after the indicated treatment (cells with or without LPS at 1 μg/mL for 24 and 48 h administration or treated with or without LY294002 at 0.5 μM for 24 h and followed by stimulation with LPS at 1 μg/mL) for ELISA analysis. Hundred microliters culture supernatant was analysed using the ELISA kits (IL‐1β, IL‐4, TNF‐α, IFN‐γ, IL‐6 and IL‐10) (Thermo Fisher Scientific, Inc.) according to the kit instructions. The Optical density was measured at 450 nm with a BioTek microplate reader.

### 2.19 Double immunofluorescence of the murine lung TCs

The paraffin slices were baked at 60°C for 1 h. After the xylene and ethanol were dehydrated, they were placed in sodium citrate solution for antigen repair. 3% H_2_O_2_ was incubated at room temperature for 5–10 min, PBS was washed two to three times, 0.3% triton × 100 was incubated at room temperature for 20 min, 10% serum was diluted with DPBS at room temperature and sealed for 1 h. The primary antibody anti‐CD34 (1:100; 0.552 mg/mL) and anti‐PDGFR‐alpha (1:500; 0.576 mg/mL), were incubated overnight, washed with PBS for three times, 5 min each time, then the fluorescent secondary antibody (goat anti‐rabbit) (1:20,000; 0.1 μg/mL) was washed at 37°C for 30 min. The antibodies mentioned were all from Abcam.

All were washed with PBS for three times, 3 min each time. DAPI was added dropwise and incubated in dark for 2 min, washed with PBS for three times, 1 min each time, 10% glycerol PBS film, and then immediately observed under fluorescence microscope.

### 2.20 Statistics

All values were showed as mean ± SEM. SPSS Statistics 24 (IBM) was used to analyse the data. The statistical differences between two groups were compared by *t*‐test. The statistical differences among over two groups were analysed by anova. *p* < 0.05 was considered statistically significant.

## 3 RESULTS

**FIGURE 1:**
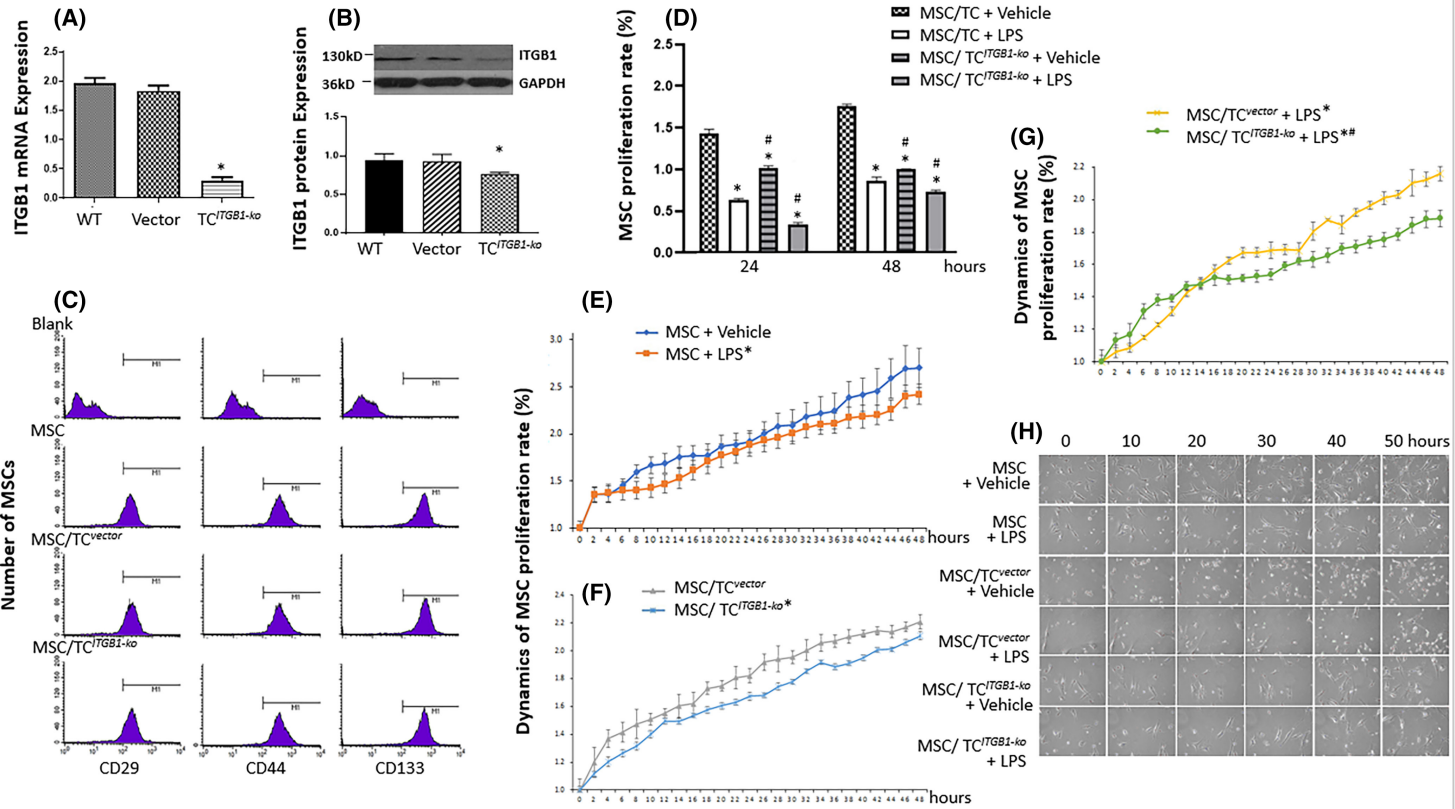
Proliferation and dynamic bio‐behaviours of MSCs co‐cultured with TC^*ITGB1‐ko*^ and lipopolysaccharide (LPS) challenged. (A) ITGB1 mRNA Expression levels of TCs and TCs with ITGB1 knock out (TC^*ITGB1‐ko*^). (B) ITGB1 Protein levels and qualification of TCs and TC^*ITGB1‐ko*^. (C) Verification of MSCs with TC^*vector*^ or TC^*ITGB1‐ko*^ by the expression of CD29, CD44 or CD133. (D) The proliferation rate of MSCs co‐cultured with TC^*vector*^ or TC^*ITGB1‐ko*^ and challenged with LPS. (E) Dynamic of MSCs proliferation rate with LPS stimulated. (F) Dynamic of MSCs proliferation rate with o‐cultured with TC^*vector*^ or TC^*ITGB1‐ko*^. (G) Dynamic of MSCs proliferation rate with o‐cultured with TC^*vector*^ or TC^*ITGB1‐ko*^ and challenged with LPS. (H) Dynamic bio‐behaviours of MSCs with o‐cultured with TC^*vector*^ or TC^*ITGB1‐ko*^ and challenged with LPS for 50 h. * stands for *p* < 0.05, as compared with MSCs challenged with vehicle, while # stands for *p* < 0.05, as compared with MSCs pretreated with LPS, at 24 h or 48 h respectively.

We firstly evaluated the quality of TC^*ITGB1‐ko*^ cells and found the expression of ITGB1 gene (Figure 1A) and protein (Figure 1B) in TC^*ITGB1‐ko*^ cells was significantly lower than in TC^*vector*^, respectively, about 80%–90% down‐regulation rate of gene. The surface markers CD29, CD44 and CD133 of MSCs were measured using flow cytometry 24 h after culture of MSCs alone (Figure 1C). The results of CCK8 assay showed that the proliferation rate of MSCs treated with LPS were decreased as compared with vehicle (Figure 1D). The proliferation rate of MSCs co‐cultured with TC^*ITGB1‐ko*^ were decreased as compared with co‐cultured with TC^*vector*^ (Figure 1D). Dynamics of MSCs proliferation rates analysis demonstrated the same tendency (Figure 1E,F). We noticed that LPS stimulation significantly reduce the proliferation rate of MSCs in MSCs alone (Figure S1A) or MSC with TC^*vector*^ or TC^*ITGB1‐ko*^ (Figure 1G), as compared with those added with vehicle (*p* < 0.05 or less). The proliferation rate of MSCs with TC^*ITGB1‐ko*^ was markedly lower than that in MSC with TC^*vector*^ pretreated with vehicle or LPS at 24 and 48 h, respectively. The proliferation rate of MSCs in MSC with TC^*ITGB1‐ko*^ challenged with LPS at 48 h was markedly higher than that at 24 h (*p* < 0.01). LPS administration inhibited dynamic proliferation of MSCs from 8 h and on (*p* < 0.05, Figure 1E; Figure S1A,B). The level of MSC proliferation dynamics in co‐culture with TC^*ITGB1‐ko*^ was significantly lower than that with TC^*vector*^ from 4 h and on (*p* < 0.05, Figure 1F; Figure S1C,D). MSC proliferation increased during 2–8 h and decreased in co‐culture with TC^*ITGB1‐ko*^ 20 h after LPS administration, as compared with MSC with TC^*vector*^, respectively (*p* < 0.05, Figure 1G). Proliferation dynamics of MSCs were also evidenced by images at different time points after MSCs with TC^*ITGB1‐ko*^ or TC^*vector*^ were challenged with LPS (Figure 1H).

**FIGURE 2:**
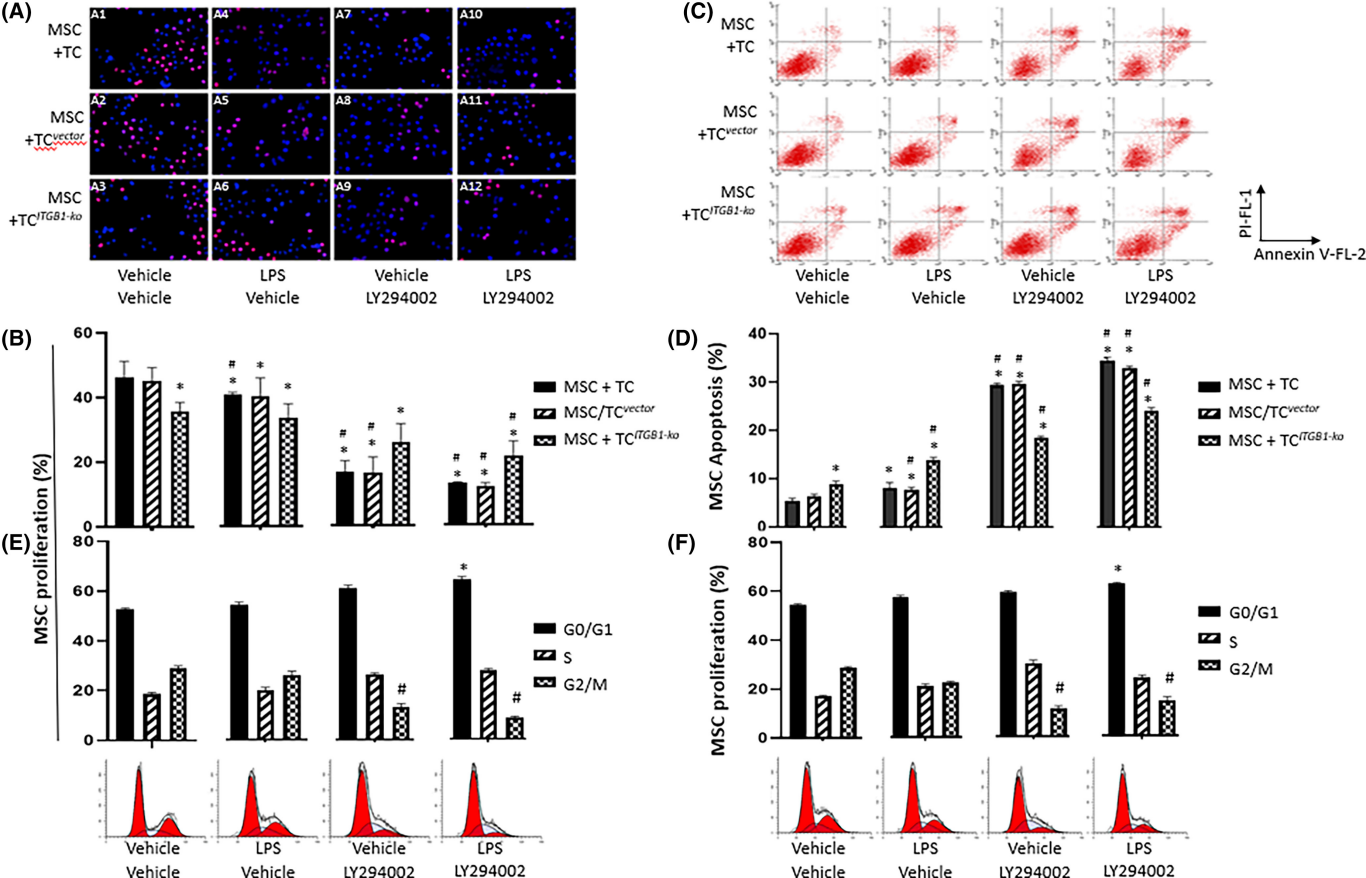
PI3K signals involved in the effect of TC^*ITGB1‐ko*^ on proliferation, apoptosis and cell cycle of MSCs with lipopolysaccharide (LPS) challenged. (A) Image of EdU labelled MSCs (red) co‐cultured with TC^*ITGB1‐ko*^ and treated with LPS or LY294002. Nucleus were tracked with Hoechst 33342(blue). (B) Quantification of MSCs proliferation of (A). (C) Apoptosis of MSC co‐cultured with TC^*ITGB1‐ko*^ and treated with LPS or LY294002. (D) Apoptosis rate of MSC. Cell cycle analysis of MSCs co‐cultured with TC^*vector*^ (E) or TC^*ITGB1‐ko*^ (F). The ratios alteration of G0/G1, S and G2/M phase were shown. * stands for *p* < 0.05, as compared with MSCs co‐cultured with TC^*vector*^ challenged with vehicle, while # stands for *p* < 0.05, as compared with MSCs co‐cultured with TC^*ITGB1‐ko*^ challenged with vehicle.

We furthermore investigated roles of PI3K in the interaction between MSCs and TCs using PI3Kα/δ/β inhibitor LY294002. Figure 2A presents images of fluorescein‐labelled MSCs and TCs. Levels of MSC proliferation in co‐culture with TC^*ITGB1‐ko*^ were significantly lower than those with TCs or TC^*vector*^ (Figure 2B). LY294002 significantly inhibited MSC proliferation, while proliferation levels of MSC with TC TC^*ITGB1‐ko*^ were significantly higher than as those with TCs or TC^*vector*^ (*p* < 0.05, respectively). There was no obvious difference in MSC proliferation between LY294002‐treated cells with LPS or vehicle. The percentage of apoptotic MSCs significantly increased in LY294002‐treated cells, as compared with vehicle‐treated cells (*p* < 0.01, respectively, Figure 2C,D; Figure S1E,F). Apoptotic number of MSCs with TC^*ITGB1‐ko*^ were significantly higher than those with TCs or TC^*vector*^ treated with vehicle, while lower than those with LY294002. LPS increased the percentage of apoptotic MSCs, as compared with vehicle. The number of MSCs with TC^*vector*^ (Figure 2E) or TC^*ITGB1‐ko*^ (Figure 2F) increased in phases of G0/G1 and S, while decreased in G2/M, after treatment with LY294002, as compared with vehicle (Figure 2E,F; Figure S2). The number of MSCs with TC^*ITGB1‐ko*^ challenged with vehicle or LPS elevated in phases of S or G2/M, as compared with those with TC^*vector*^, respectively.

**FIGURE 3:**
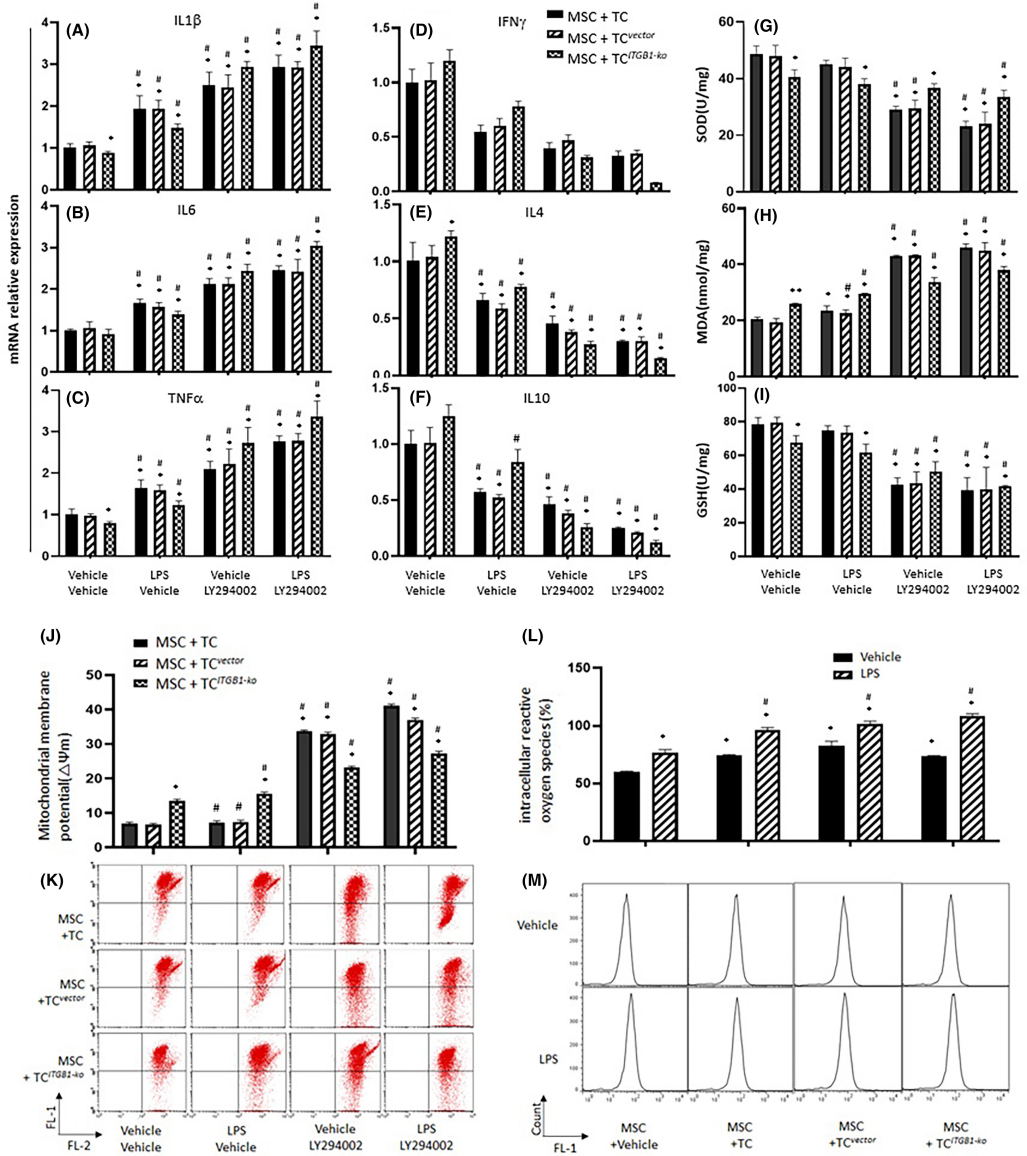
Potential factors, oxidative stress indexes and mitochondrial membrane potential of MSCs co‐cultured with TC^*ITGB1‐ko*^ and lipopolysaccharide (LPS) challenged. IL‐1β (A), IL‐6 (B), TNF‐α (C), IFN‐γ (D), IL‐4 (E), IL‐10 (F) mRNA levels of MSCs were detected after co‐cultured with TC^*ITGB1‐ko*^. Oxidative stress was detected with SOD (G), MDA (H), GSH (I). (J, K) JC‐1 was detected by flow cytometry and analysed on MSCs co‐cultured with TC^*ITGB1‐ko*^ and LPS challenged. * stands for *p* < 0.05, as compared with MSCs co‐cultured with TC^*vector*^ challenged with vehicle, # stands for *p* < 0.05, as compared with MSCs co‐cultured with TC^*ITGB1‐ko*^ challenged with vehicle. (L, M) ROS was detected by flow cytometry on MSCs co‐cultured with TC, TC^*vector*^ and TC^*ITGB1‐ko*^. * stands for *p* < 0.05, as compared with MSCs challenged with vehicle, while # stands for *p* < 0.05, as compared with MSCs pretreated with LPS.

Levels of inflammatory factors such as IL‐1β (Figure 3A), IL‐6 (Figure 3B) and TNF‐α (Figure 3C) produced from MSCs with TCs, TC^*vector*^ or TC^*ITGB1‐ko*^ challenged with LPS were markedly higher than those with vehicle. Levels of IL‐1β, IL‐6 and TNF‐α in MSCs with TC^*ITGB1‐ko*^ treated with vehicle were lower than those in MSCs with TC^*vector*^, while higher than those treated with LY294002. Levels of TNF‐α, IL‐6 and IL‐1β in MSCs treated with LY294002 were significantly higher, as compared with those with vehicle. In opposite, levels of IFN‐γ (Figure 3D), IL‐4 (Figure 3E) and IL‐10 (Figure 3F) decreased in MSCs with TCs, TC^*vector*^ or TC^*ITGB1‐ko*^ challenged with LPS or treated with LY294002, as compared with vehicle. Levels of IFN‐γ, IL‐4 and IL‐10 in MSC with TC^*ITGB1‐ko*^ treated with vehicle were significantly higher than those with TCs or TC^*vector*^, or while lower than in those treated with LY294002 (*p* < 0.05 or less, respectively, Figure 3). Levels of IL‐1β, IL‐6 and TNF‐α produced from MSCs with TCs, TC^*vector*^ or TC^*ITGB1‐ko*^ challenged with LPS were significantly higher than those with vehicle. Levels of IL‐6, IL‐1β and TNF‐α in MSCs with TC^*ITGB1‐ko*^ treated with vehicle were lower than those in MSCs with TC^*vector*^, while higher than those treated with LY294002. Levels of IL‐6, IL‐1β and TNF‐α in MSCs treated with LY294002 were significantly higher, as compared with those with vehicle. In opposite, levels of IFN‐γ, IL‐4 and IL‐10 decreased in MSCs with TCs, TC^*vector*^ or TC^*ITGB1‐ko*^ challenged with LPS or treated with LY294002, as compared with vehicle. Levels of IFN‐γ, IL‐4 and IL‐10 in MSC with TC^*ITGB1‐ko*^ treated with vehicle were significantly higher than those with TCs or TC^*vector*^, or while lower than in those treated with LY294002 (*p* < 0.05 or less, respectively, Figure S3). Levels of SOD decreased significantly in MSCs with TCs or TC^*vector*^ treated with LY294002, rather than those with TC^*ITGB1‐ko*^ (Figure 3G). SOD levels in MSCs with TC^*ITGB1‐ko*^ were lower than those with TCs or TC^*vector*^ with vehicle, while higher in those with LY294002. Levels of MDA were significantly higher in MSCs with TCs, TC^*vector*^ or TC^*ITGB1‐ko*^ with LY294002, as compared with those with vehicle (Figure 3H), while GSH was higher in TC^*ITGB1‐ko*^ treated with vehicle and lower in MSCs with TC^*ITGB1‐ko*^ with LY294002, as compared with those with TCs or TC^*vector*^ (Figure 3I). Levels of mitochondrial membrane potentials of MSCs co‐cultured with TC^*ITGB1‐ko*^ were significantly higher than those of MSCs with TC^*vector*^ after LPS challenge (Figure 3J,K), while lower after LPS in LY294002 condition (*p* < 0.05 or less). Levels of intracellular reactive oxygen increased significantly species in MSCs co‐cultured with or without TCs treated with LPS, as compared with those with vehicle, and were higher in MSCs co‐cultured with TCs, TC^*vector*^ or TC^*ITGB1‐ko*^ than those in MSCs alone (Figure 3L,M).

**FIGURE 4:**
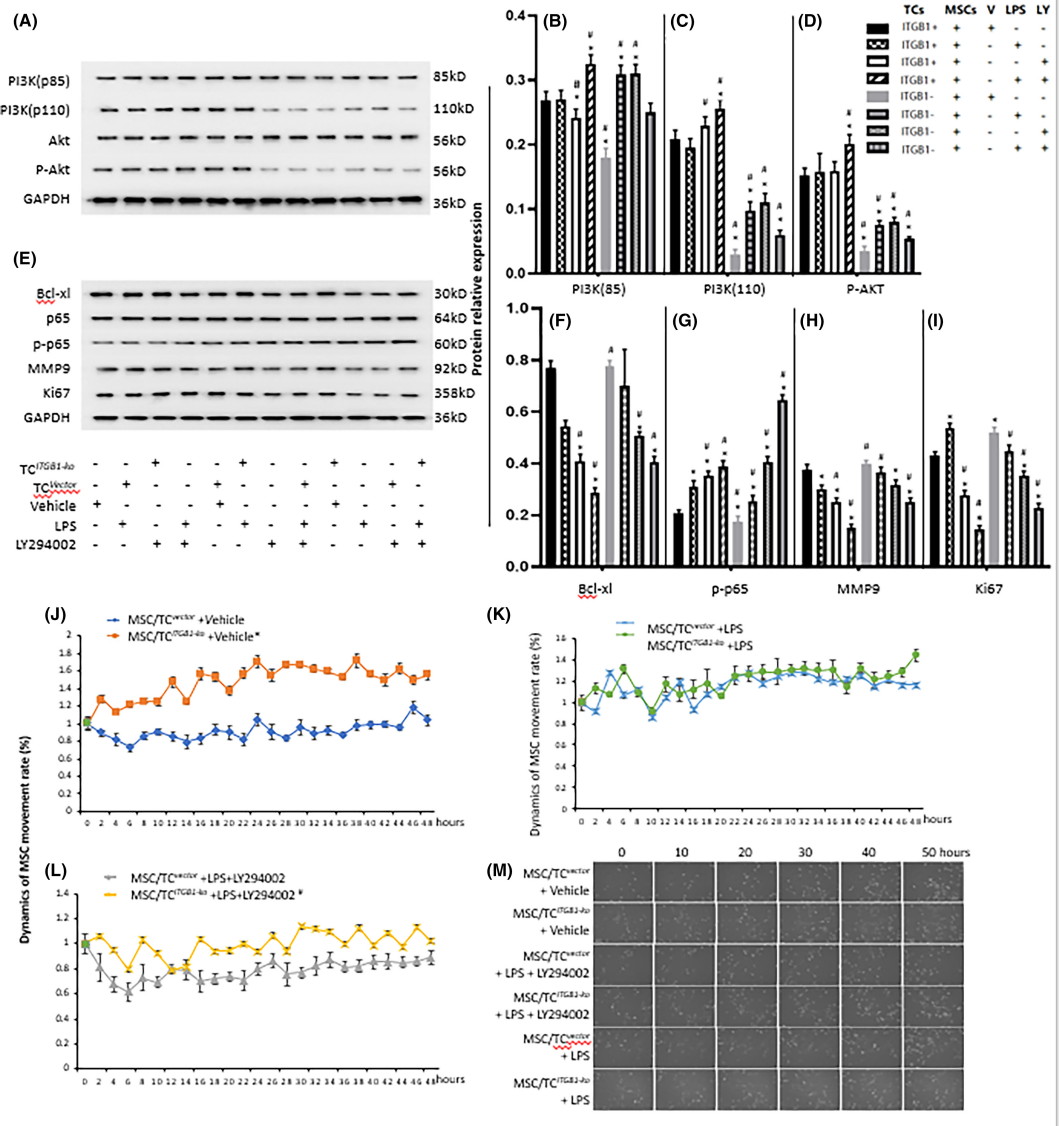
PI3K signals involved in the effect of TC^*ITGB1‐ko*^ on MSCs with lipopolysaccharide (LPS) challenged and influence of TC^*ITGB1‐ko*^ on MSCs bio‐behaviours. (A) Protein expression of PI3K p110, PI3K p85, AKT and p‐AKT of MSCs were detected by Western Blot. Quantification analysis of PI3K p110 (A), PI3K p85 (B), AKT (C), p‐AKT (D) of MSCs were shown. (E) Protein band of Bcl‐xl, p65, p‐p65, MMP9, Ki67 of MSCs. Quantification analysis of Bcl‐xl (F), p‐p65 (G), MMP9 (H), Ki67 (I) of MSCs were shown. * stands for *p* < 0.05, as compared with MSCs co‐cultured with TC^*vector*^ challenged with vehicle, # stands for *p* < 0.05, as compared with MSCs co‐cultured with TC^*vector*^ challenged with LPS. GAPDH was used as internal control. Dynamics of MSCs movement (J–M) was monitored using the Cell‐iq system. Cell movement of MSCs co‐cultured with TC, TC^*vector*^ or TC^*ITGB1‐ko*^ challenged with vehicle (V) or LPS pretreated with LY294002 were detected. (M) Images of MSCs movement after treatment for 0, 10, 20, 30, 40 and 50 h. * stands for *p* < 0.01, as compared with MSCs co‐cultured with TC^*vector*^ challenged with vehicle, # stands for *p* < 0.05, as compared with MSCs co‐cultured with TC^*vector*^ challenged with LPS and LY294002.

Figure 4A demonstrates levels of PI3K p85, PI3K p110, AKT and p‐AKT in MSCs with TC^*vector*^ or TC^*ITGB1‐ko*^ challenged with vehicle or LPS and treated with vehicle or LY294002. Levels of PI3K p85 proteins were lower in MSCs with TC^*vector*^ with LY294002 than those with vehicle, and significantly higher in those treated with LPS and LY294002, as compared with those treated with vehicle or challenged with LPS, respectively (*p* < 0.01, Figure 4B). Levels of PI3K p85 in MSCs with TC^*ITGB1‐ko*^ treated with LPS were markedly higher than those with vehicle, while in MSCs with TC^*ITGB1‐ko*^ with LPS and LY294002 were lower than those with LPS alone. Levels of PI3K p85 in MSCs with TC^*ITGB1‐ko*^ were significantly lower than those with TC^*vector*^, while in MSCs with TC^*ITGB1‐ko*^ challenged with LPS were higher than those with MSCs with TC^*ITGB1‐ko*^ challenged with vehicle or with TC^*vector*^ with vehicle or LPS. Levels of PI3K p110 (Figure 4C) and p‐AKT (Figure 4D) in MSCs with TC^*ITGB1‐ko*^ with or without LPS or LY294002 were significantly lower than those with TC^*vector*^ (*p* < 0.05 or less, respectively). Figure 4E shows protein levels of Bcl‐xl, p65, phosphorylated p65(p‐p65), MMP9, and Ki67 in MSCs with TC^*vector*^ or TC^*ITGB1‐ko*^ pretreated with vehicle, LPS or LY294002. LPS reduced levels of Bcl‐xl (Figure 4F), MMP9 (Figure 4H), and Ki67 (Figure 4I) in MSCs with TC^*vector*^ or TC^*ITGB1‐ko*^ treated with vehicle or LY294002, as compared with the corresponding cells with vehicle, except for Ki67 protein in MSCs with TC^*vector*^ with LPS (Figure 4I). Levels of those proteins in MSCs with TC^*vector*^ or TC^*ITGB1‐ko*^ treated with LY294002 were markedly lower than those with vehicle. Levels of p‐p65 increased in MSCs with TC^*vector*^ or TC^*ITGB1‐ko*^ with LPS and were even higher after treatment with LY294002 (Figure 4G). Figure 4J–M demonstrates levels of MSC movement dynamics monitored and recorded at interval each 2 h for 24 h. We found that levels of movement dynamics of MSCs with TC^*ITGB1‐ko*^ were markedly higher than that of MSCs with TC^*vector*^ (*p* < 0.01, Figure 4J). There was no difference of movement dynamics between MSCs with TC^*vector*^ or TC^*ITGB1‐ko*^ treated with LPS (Figure 4K). The movement dynamics of MSCs with TC^*ITGB1‐ko*^ was higher than TC^*vector*^ in response to both LPS and LY294002 (Figure 4L). The images of MSC movements in details are shown in Figure 4M.

**FIGURE 5:**
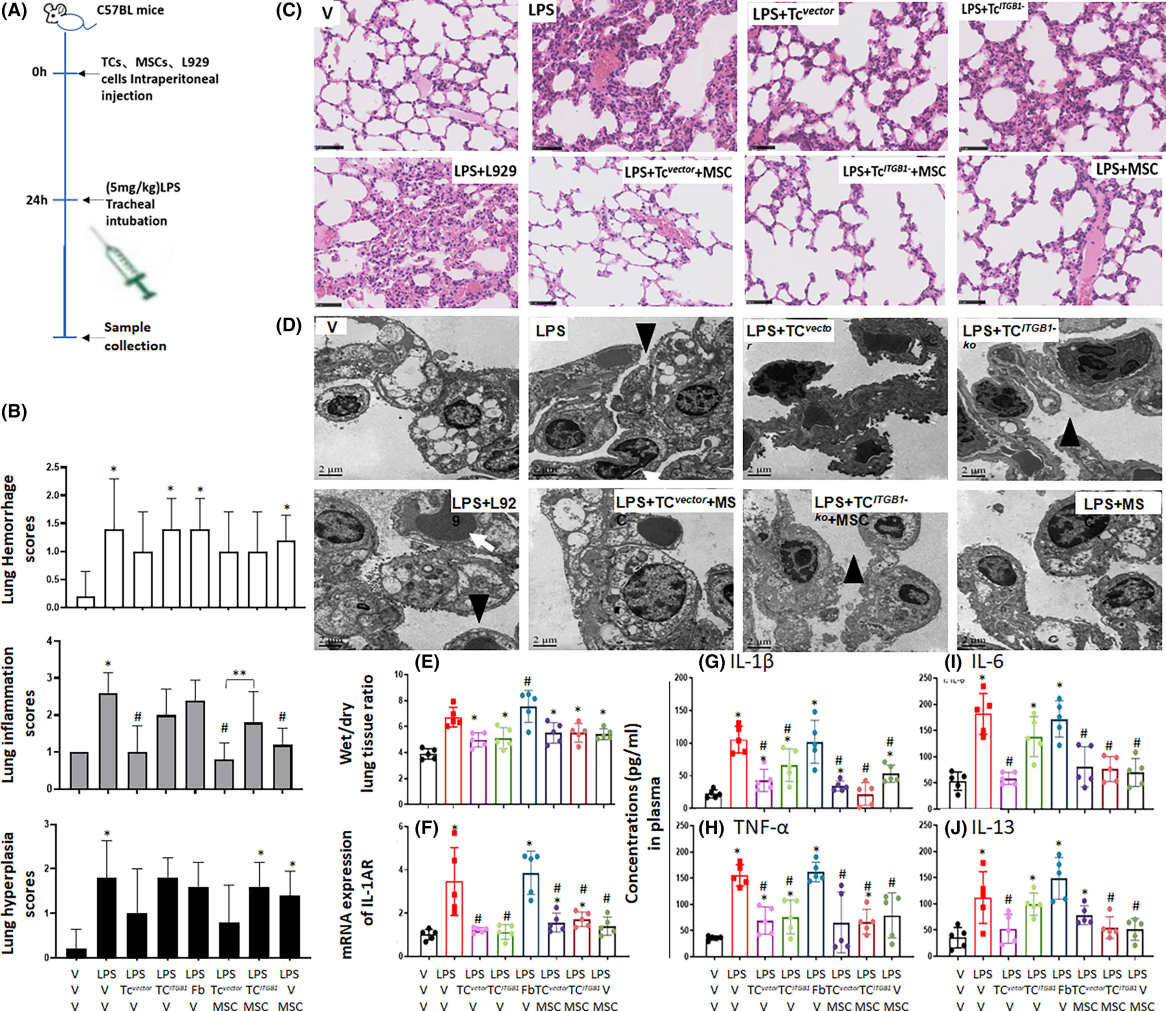
Preventive roles of mouse lung‐derived telocytes (TCs) in experimental acute lung injury induced by lipopolysaccharide (LPS). (A) experimental workflow where mice were pretreated and challenged with vehicle or LPS and TCs, MSCs, L929 cells. (B) Pathological scores of lung tissue haemorrhage, inflammation, and hyperplasia of every group were analysed. (C) Pathological changes of lung tissues harvested from mice and stained with Haematoxylin and eosin at ×100 (up panel) and ×200 (low panel), respectively. (D) Paragraphs of lung ultrastructure by TEM (10, 000×). Mice were pretreated and challenged with vehicle (V), pretreated with vehicle and challenged with LPS (LPS), pretreated with TC^*vector*^ and challenged LPS (LPS + TC^*vector*^), pretreated with TC^*ITGB1‐ko*^ and challenged LPS (LPS + TC^*ITGB1‐ko*^), pretreated with L929 cells (LPS + L929), pretreated with TC^*vector*^, MSC and challenged LPS (LPS + TC^*vector*^ + MSC), pretreated with TC^*ITGB1‐ko*^ and challenged LPS (LPS + TC^*ITGB1‐ko*^ + MSC), or pretreated with MSC and challenged LPS (LPS + MSC) (*n* = 5/group). Black arrow head showed alveolar pneumocytes and white arrow head showed migrated leukocytes. (E) Radio of Wet/dry lung weight. (F) mRNA expression of interleukin (IL) 1RA in lung tissues. (G) Plasma levels of IL‐1β. (H) Plasma levels of TNF‐α. (I) Plasma levels of IL‐6. (J) Plasma levels of IL‐13. Mice were pretreated with TCs, MSCs or L929 cells at 10^6^ intraperitoneally 24 h prior to intratracheal administration of LPS. * stands for *p* < 0.05, as compared with mice challenged with vehicle, # stands for *p* < 0.05, as compared with mice challenged with LPS, ** stands for *p* < 0.05, as mice pretreated with TC^*ITGB1‐ko*^ and MSCs compared with mice pretreated with TC^*vector*^ and MSCs challenged with LPS.

Experimental acute lung injury can be induced by intratracheal instillation of LPS, of protocol schedules and manipulations are shown in Figure 5A. LPS increased significantly levels of acute lung histological scores in animals without cell therapy, as compared with those challenged with vehicle (*p* < 0.05, Figure 5B), which was significantly prevented by co‐transplantation of MSCs with TC^*vector*^ (*p* < 0.05). The CD34 animals with LPS (Figure 5C), as compared with those with vehicle. The severity of lung injury induced by LPS in animals with fibroblasts was similar to those in animals without cell therapies. The intraperitoneal instillation of TC^*vector*^, TC^*ITGB1‐ko*^ or MSCs alone or MSCs with TC^*vector*^ or TC^*ITGB1‐ko*^ improved the severity of LPS‐induced acute lung injury, as compared with those without cell therapy or with fibroblasts. The severity of lung injury in animals with TC^*vector*^ or MSCs with TC^*vector*^ was better than those in animals with TC^*ITGB1‐ko*^ or MSCs with TC^*ITGB1‐ko*^, respectively. More appearance of red blood cells within the lung interstitial space was observed in animals with MSCs alone or MSCs with TC^*ITGB1‐ko*^, as compared with those with MSCs with TC^*vector*^ (Figure 5C). Lung distribution of TCs were identified after the intravenous injection with the special cellular surface biomarkers CD34 and PDGFRα in mice lung tissues of LPS‐induced acute lung injury or transplanted with TC^*vector*^, TC^*ITGB1‐ko*^, or MSCs alone or MSCs with TC^*vector*^ or TC^*ITGB1‐ko*^ with the challenge of LPS (Figure S5A). TEM tomography also showed that TCs have narrow and flat cellular prolongations surrounding other TCs. Mitochondria and telopodes (Tps) which is one of characteristic structures of were shown as well (Figure S5B).

We found that the intercellular connections among alveolar pneumocytes were broken and more leukocytes migrated through broken barriers (Figure 5D), as compared with the intact connections between alveolar epithelial cells of animals without LPS. The alveolar barriers of animals with TC^*vector*^ alone were more intact than those with TC^*ITGB1‐ko*^ alone after LPS challenge. The integrity of alveolar connections became loose and particles were leaked into alveolar cavities in animals with LPS and fibroblast implant. The tight junctions and contacts between alveolar epithelia were intact in LPS‐challenged animals implanted with MSCs and TC^*vector*^, as compared with those implanted with TC^*vector*^ or TC^*ITGB1‐ko*^ alone, with MSCs and TC^*ITGB1‐ko*^, or with MSCs alone. The severity of acute lung oedema estimated by the ratio of lung tissue dry and wet weights significantly increased animals challenged with LPS without cell therapy or with fibroblast therapy, as compared with those without challenge, or with LPS and intraperitoneal injection of TC^*vector*^, TC^*ITGB1‐ko*^, or MSCs alone, or MSCs with TC^*vector*^ or TC^*ITGB1‐ko*^ (Figure 5E). Expressions of IL‐1RA mRNA (Figure 5F) as well as protein levels of IL‐1β (Figure 5G), TNF‐α (Figure 5H), IL‐6 (Figure 5I) and IL‐13 (Figure 5J) in plasma significantly increased in animals challenged with LPS without cell therapy or with fibroblast therapy. However, the mRNA levels of IL‐10, Angiopoietin‐1 (Ang‐1), IL‐1β, TNF‐α, IL‐1RA and IL‐13 in lung tissue were not changed significantly (Figure S4).

## 4 DISCUSSION

Our previous studies demonstrated that combined transplantation of TCs and MSCs at the combination of high doses could alleviate about 50% of LPS‐induced acute lung injury, while mono‐transplantation of TCs or MSCs only reached about 10%–20% inhibitory effects.^19^ The injection of conditional mediums from MSCs and TCs co‐culture at high concentration of both cells showed about 15%–20% inhibitory effects. Co‐transplanted TCs with MSCs were observed in lung tissue of LPS induced mice models.^18^, ^19^ Those data indicates that TCs have supportive or nutritional effects on MSCs directly and indirectly through the activation of serial signaling pathways, including ITGB1‐, RBP1‐, GJA1‐, FGF10‐, EGF‐ and VEGFβ‐dominated networks.

ITGB1‐orientated networks mainly contain ITGB1, OPN, ITGA4, CD44, ITGB3, ITGA9, THBS2, ITGA, MMP3 and MMP7. Of those heterodimeric adhesion receptors for extracellular matrices, the α subunit can heterodimerize with the β1, β3, β5, β6 or β8 subunits, while β1 with α subunits from α1 to α11, and αv. Song et al.^14^ found that TCs communicated with other cells through TGFβ1‐ITGB1‐PI3K signaling pathways especially PI3Kp110α, PI3Kα/δ, PKCβ or GSK3, responsible for cell–cell communication, cell‐extracellular matrix and cell–cell adhesion. The cell–cell communication is the decisive process for the signalling and function between cells within the microenvironment, through initiator cells, signal network factors, communicating conditions and receptor cells.^14^ Of important signaling pathways, PI3K‐associated and dependent signals play critical roles in the activation of cell–cell communication and the maintenance of tissue integrity and function.^11^ AlMusawi et al.^1^ demonstrated that patterns and modes of inter‐cellular signal transmissions and interplay among the same or different cell‐types shaped the function and role of tissue microenvironment in organ development or tumorigenesis. The present study investigates molecular mechanisms of TCs‐MSCs interactions with a special focus on the role of TCs‐driven ITGB1 in function and therapeutic potential of MSCs in inflammatory condition. Before the study, triple immunofluorescent staining for CD34, PDGFRα and vimentin was performed to conform the TCs cell line stability (Figure S6).

ITGB1, also known as CD29 a member of integrin β subfamily, contributes to cell signal, adhesion, growth, cycle, differentiation, migration and survival, and to early development, haematopoiesis, tumorigenesis and assembly of extracellular matrix proteins through regulation of protein binding and heterodimerization, and receptor‐mediated activity.^12^ ITGB1 also contributes to the quality control of the tissue microenvironment, homeostasis and repair and to the maintenance of cell biological phenomes within the microenvironment by regulating the interaction between resident cells with the extracellular matrix. Our previous study first demonstrated the heterogeneity of network characters and molecular interactions of integrin family genes among lung TCs with other tissue cells resident in the lung, for example airway basal cells, MSCs, fibroblasts, alveolar type II cells, proximal airway cells, CD8^+^ T cells from bronchial lymph nodes and lungs.^15^ The intracellular activation of ITGB1‐TGFβ1‐PI3K signaling pathways could play important roles in cell survival, proliferation, and sensitivity to PI3K subunit signals. ITGB1‐orentiated networks were proposed as one of the major regulator network panels during the interaction of TCs‐MSCs in the inflammatory condition.^19^ We hypothesize that TCs‐driven ITGB1 play important roles in the interaction and communication between TCs and MSCs, in the support and benefit to MSCs, and in the improvement of MSCs function and activity. The present results showed that the ITGB1‐dominated signals play critical roles in TC‐associated cell–cell communication, evidenced by the fact that the down‐regulation of ITGB1 near 90% inhibition of ITGB1 gene expression in TCs led to the reduction of TC‐supportive effects on proliferation dynamics of MSCs. TCs^*ITGB1‐ko*^ loss the capacity of supporting MSC proliferation, while the partial effects of TC^*ITGB1‐ko*^ on some biological phenomes of MSCs might be probably associated with the partial reduction of ITGB1 proteins in TC^*ITGB1‐ko*^ or diverse roles of TC integrin receptors in MSCs in different conditions. Our previous studies demonstrated that combined transplantation of TCs and MSCs could prevent and treat experimental lung tissue inflammation which is consistent with our previous studies. Figure 5 shows that lung inflammation score of transplantation with TC^*vector*^ + MSCs for treatment was lower than transplantation with TC^*vector*^ or MSCs for treatment alone, however without significance. Our current studies showed the co‐transplantation of MSCs and TCs or TCs^*ITGB1‐ko*^ on acute lung injury induced by LPS had preventive therapeutic effects. Figure 5 shows co‐transplantation of TC^*vector*^ and MSCs could decrease the inflammation score than mono‐transplantation, but without significance. However, the significant elevation of lung inflammation score in co‐transplantation with TC^*ITGB1‐ko*^ and MSCs could state the function of ITGB1 in lung tissue inflammation treatment. ITGB1‐orentiated networks were proposed as one of the major regulator network panels during the interaction of TCs‐MSCs in the inflammatory condition.^19^ Lung inflammation score of co‐transplantation with TC^*ITGB1‐ko*^ and MSCs was significantly higher than transplantation with TC^*vector*^ or MSCs alone for treatment (Figure 5B) which demonstrated the function of ITGB1 in transplantation of TCs‐MSCs in experimental lung tissue inflammation treatment.

**FIGURE 6:**
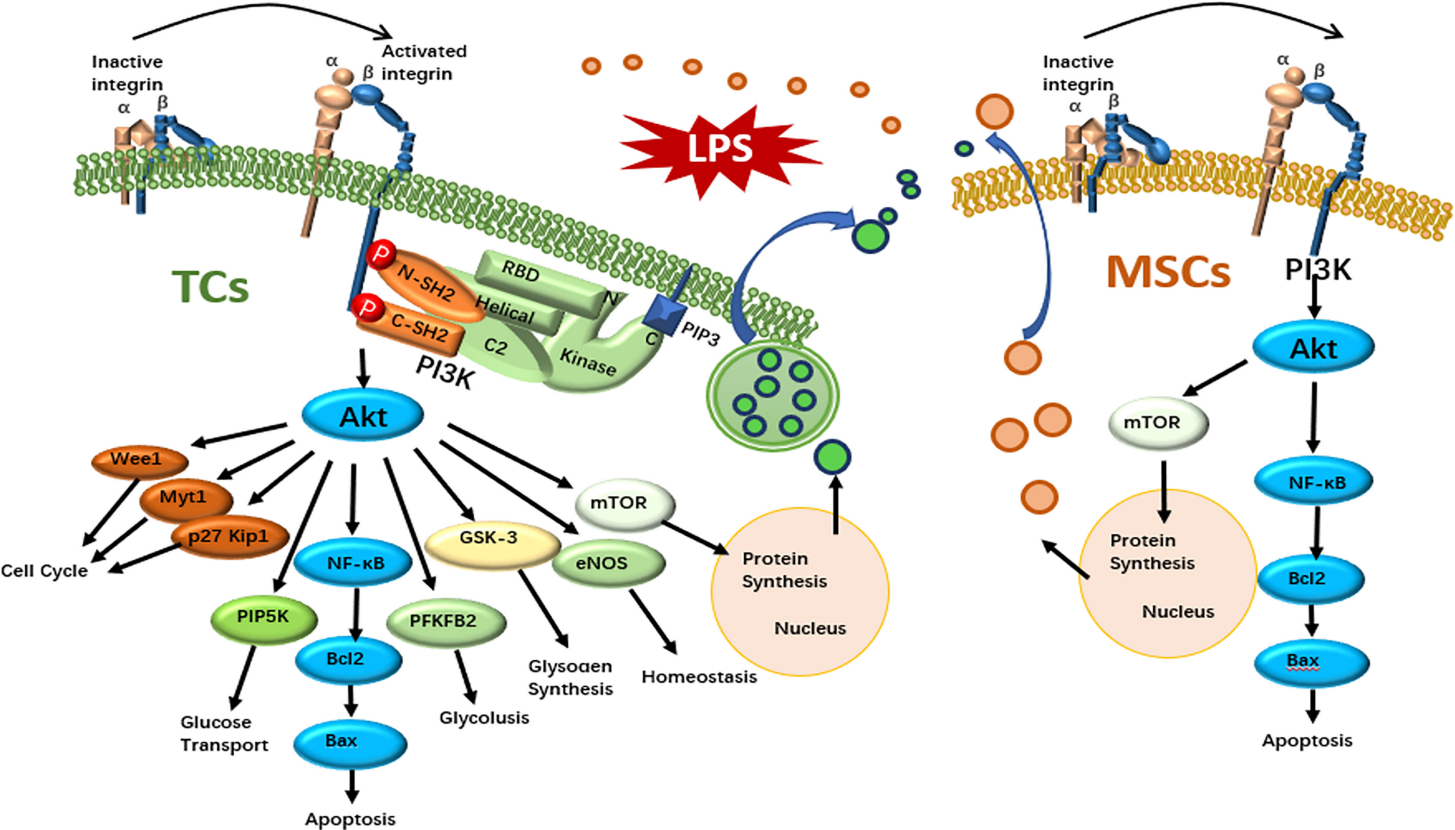
Schematic diagram of processing telocytes (TCs) and mesenchymal stem cells (MSCs). The pathway map of the interaction between TC and MSC stimulated by lipopolysaccharide (LPS).

The interactions and signal transmission circuits between ITGB1 and PI3K maintain cellular phenomes and functions. ITGB1 regulates cell integrity, shape, migration, proliferation through PI3K signaling pathways. Our previous data demonstrated that the deletion of ITGB1 could change TCs sensitivities and responses to TGF‐β1 and pan‐PI3K, PKC, PI3K p110α and GSK3β inhibitors.^14^ Our present study furthermore investigated roles of TC^*ITGB1‐ko*^ in bio‐behaviours of MSCs and found that TC^*ITGB1‐ko*^ co‐culture altered the sensitivity of MSCs to PI3Kα/δ/β inhibitor. Gathering the information, the deletion of ITGB1 in TCs influences cell phenomes and functions not only for TCs per se, but also for TC‐communicated other cells. ITGB1 in TCs plays a decisive role in the maintenance of MSC PI3K p85 sensitivity to LPS challenge or LY294002, although the exact mechanisms by which ITGB1 and PI3K interact remain unclear. The ITGB1/PI3K signaling pathway also contributes to the process of stem cell‐driven wound healing, development of drug resistance, tumorigenesis and progression, mature of vaccination and immune metabolism and cell adhesions. Guo et al.^4^ found that thrombospondin 4 as an oncogene regulated the development of the epithelial‐mesenchymal transition and interacted with ITGB1 to modulate FAK/PI3K/AKT pathway. The present studies investigated potential molecular mechanisms of interactions between TCs and MSCs with a focus on ITGB1 in TCs. LY294002 is a potent and selective PI3K inhibitor, and TC ^ITGB1‐ko^ co‐culture altered the sensitivity of MSCs to PI3K inhibitor. Thus, the use of LY294002 can increase the expression of the active forms of PI3K and pAKT in MSC/ ITGB1‐delated TCs. Of those mediators, long noncoding RNA or microRNA can initiate and activate cancer development by ITGB1/FAK/PI3K/AKT signals. We found that changes of MSC proliferation and apoptosis were accompanied with the dysregulation of cytokine mRNA expression in MSCs co‐cultured with ITGB1‐deleted TCs during the exposure of PI3Kα/δ/β inhibitor, of which IL‐1β, IL‐6, and TNF‐α increased, while IFN‐γ, IL‐4 and IL‐10 decreased. It indicates that the interaction of TC^*ITGB1‐ko*^ with MSCs altered the capacity of MSCs responses to inflammation and regulators (Figure 6).

The PI3K p85/PI3K p110/pAKT were found to be important elements of ITGB1/PI3K signaling pathways, probably responsible for roles of TC^*ITGB1‐ko*^ in alterations of MSC biological phenomes and function in response to inflammation or PI3K inhibitor, although the exact mechanisms remain unclear. PI3K‐p85 and PI3K110 are the regulatory subunit (p85) with SH2 and SH3 domains and catalytic subunit (P110) with α, β, δ and γ isoforms of PI3K, which can be therapeutic targets for diseases.^3^ PI3K‐p85 can interact with target proteins through the corresponding binding sites and integrate with PI3K p110 to induce the production of second messenger PIP3 on the plasma membrane, to promote the phosphorylation of ser308 of AKT protein by binding to AKT and phosphoinositide‐dependent kinase‐1. We found that the responses of PI3K p110, PI3K p85 and pAKT of MSCs co‐cultured with ITGB1‐deleted TCs to LPS or PI3K inhibitor were opposite to those with ITGB1‐presented TCs. The levels of PI3K p110 and pAKT proteins in co‐cultured with ITGB1‐deleted TCs were obviously lower than those in ITGB1‐presented TCs. Potla et al.^8^ demonstrated that mechanical force passed through the surface of endothelial cells β1 integrin receptors delivery could activate the transmembrane solute carrier family 3 member 2 protein and the transient receptor potential vanilloid 4 ion channels. It is possible that the deletion of ITGB1 in TCs may change the mechanical forces of TCs surface membrane, fail to transmit membrane forces from the cytoplasmic C terminus, and loss the capacity of force‐induced channel activation, leading to the abnormity of inter‐communication between MSCs and TCs and the altered responses of MSCs to LPS or PI3K inhibitor. It is also possible that TCs may interact with MSCs differently due to changes of the molecular machinery and function of glycosylphosphatidylinositol‐anchored protein nanoclustering resulted from the failure of Arg‐Gly‐Asp motif‐containing ligand binding.^5^

We furthermore investigated the potential roles of ITGB1‐deleted TCs in MSCs in experimental acute lung inflammation characterized by cytokine production and leukocyte infiltration, lung tissue oedema by endothelial barrier dysfunction, and lung tissue injury by structural damage. The intraperitoneal injection of TC^*ITGB1‐ko*^, TC^*vector*^ or MSCs alone, as well as the combination of MSCs with TC^*ITGB1‐ko*^ or TC^*vector*^ exhibited therapeutic effects on LPS‐induced acute lung injury, as compared with the intraperitoneal injection of fibroblasts alone as a non‐specific functional cell control. There were no statistical differences between co‐transplantations with TC^*ITGB1‐ko*^ or TC^*vector*^, although effects of TC^*ITGB1‐ko*^ on MSCs phenomes and functions were clearly noticed in the in vitro evaluation system. One of potential explanations is that roles of ITGB1‐deleted TCs in MSCs may be rapidly compensated by animal‐inherent TCs with ITGB1, since TCs broadly exist all organs/tissues^2^ and ITGB1 expression varies among organs/tissues.^16^ Other possibility is that the mixture solution of ITGB1‐deleted TCs and MSCs had uncontrolled and little opportunities to interact and inter‐communicate each other after the intraperitoneal injection. The integrins per se can regulate the development of acute vascular permeability and leukocyte recruitment, especially at the onset of the acute inflammatory response.^9^ A possibility is that the amount of ITGB1‐deleted TCs may be insufficient to perform biological function in the in vivo system.

In conclusion, the present studies investigated potential molecular mechanisms of interactions between TCs and MSCs with a focus on integrin beta1 (ITGB1) in TCs (Figure 6). We found that the co‐culture of MSCs with ITGB1‐deleted TCs (TC^*ITGB1‐ko*^) changed the capacity of MSC proliferation, differentiation, and growth dynamics in responses to LPS or PI3K inhibitor. Changes of MSC proliferation and apoptosis were accompanied with the dysregulation of cytokine mRNA expression in MSCs co‐cultured with TC^*ITGB1‐ko*^ during the exposure of PI3Kα/δ/β inhibitor, of which IL‐1β, IL‐6 and TNF‐α increased, while IFN‐γ, IL‐4 and IL‐10 decreased. The responses of PI3K p85, PI3K p110 and pAKT of MSCs co‐cultured with TC^*ITGB1‐ko*^ to LPS or PI3K inhibitor were opposite to those with ITGB1‐presented TCs. The intraperitoneal injection of TC^*ITGB1‐ko*^, TC^*vector*^ or MSCs alone, as well as the combination of MSCs with TC^*ITGB1‐ko*^ or TC^*vector*^ exhibited therapeutic effects on LPS‐induced acute lung injury. Thus, our data indicate that telocyte ITGB1 contributes to the interaction and intercellular communication between MSCs and TCs, responsible for influencing other cell phenomes and functions.

### Perspectives

The therapeutic effect of primary TCs in in vivo models of LPS‐induced acute lung injury had been demonstrated for several times in our previous studies. As the comments mentioned, in perspective, to translate the findings to set up telocyte‐based therapeutic approaches for human disorders. Since the isolation, identification, culture and amplification in vitro of TCs is quite difficult, expensive and time‐consuming, TC‐SV40 has been constructed for future translational medicine application. Our current study is to demonstrate the therapeutic roles of TCs and co‐transplantation of TCs and MSCs, and expound the mechanisms of ITGB1 in co‐transplantation of TCs and MSCs.

## AUTHOR CONTRIBUTIONS

**Ruixue Qi:** Conceptualization (equal); resources (equal); software (equal); validation (equal); visualization (equal); writing – original draft (equal). **Jiayun Hou:** Data curation (equal); software (equal); visualization (equal). **Ying Yang:** Data curation (equal); formal analysis (equal); software (equal); supervision (equal). **Zhicheng Yang:** Formal analysis (equal); resources (equal); supervision (equal); validation (equal). **Lihong Wu:** Resources (equal); software (equal). **Tiankui Qiao:** Investigation (equal). **Xiangdong Wang:** Conceptualization (equal); funding acquisition (equal); investigation (equal); project administration (equal); visualization (equal); writing – original draft (equal); writing – review and editing (equal). **Dongli Song:** Conceptualization (equal); funding acquisition (equal); investigation (equal); project administration (equal); visualization (equal); writing – original draft (equal); writing – review and editing (equal).

## FUNDING INFORMATION

The work was supported by The Shanghai Committee of Science and Technology (21140902600, 21ZR1412800, 21140902601, 12JC1402200, 12431900207, 11410708600, 14431905100, 20JC1418200), Zhongshan Distinguished Professor Grant (XDW), The National Nature Science Foundation of China (81873409, 81700008, 91230204, 81270099, 81320108001, 81270131, 81300010). Original Research Personalized Support Project of Fudan University (IDF152064/011). Operation funding of Shanghai Institute of Clinical Bioinformatics, Ministry of Education for Academic Special Science and Research Foundation for PhD Education (20130071110043), and National Key Research and Development Program (2016YFC0902400, 2017YFSF090207, 2017YFC0909500). Shanghai Engineering Research Center of Tumour Multi‐Target Gene Diagnosis (20DZ2254300), and Key Subject Construction Program of Shanghai Health Administrative Authority (ZK2019B30).

## CONFLICT OF INTEREST STATEMENT

The authors declare that they have no competing interests.

## Supporting information


Figure S1.

Figure S2.

Figure S3.

Figure S4.

Figure S5.

Figure S6.

Table S1.

## Data Availability

The data that support the findings of this study are available from the corresponding author upon reasonable request.
