# Supplementary figures and images for: Integrin beta1 mediates the effect of telocytes on mesenchymal stem cell proliferation and migration in the treatment of acute lung injury
Source: J Cell Mol Med. 2023 Oct 19;27(24):3980–94. doi: 10.1111/jcmm.17976 (PMC10746951; doi:10.1111/jcmm.17976)

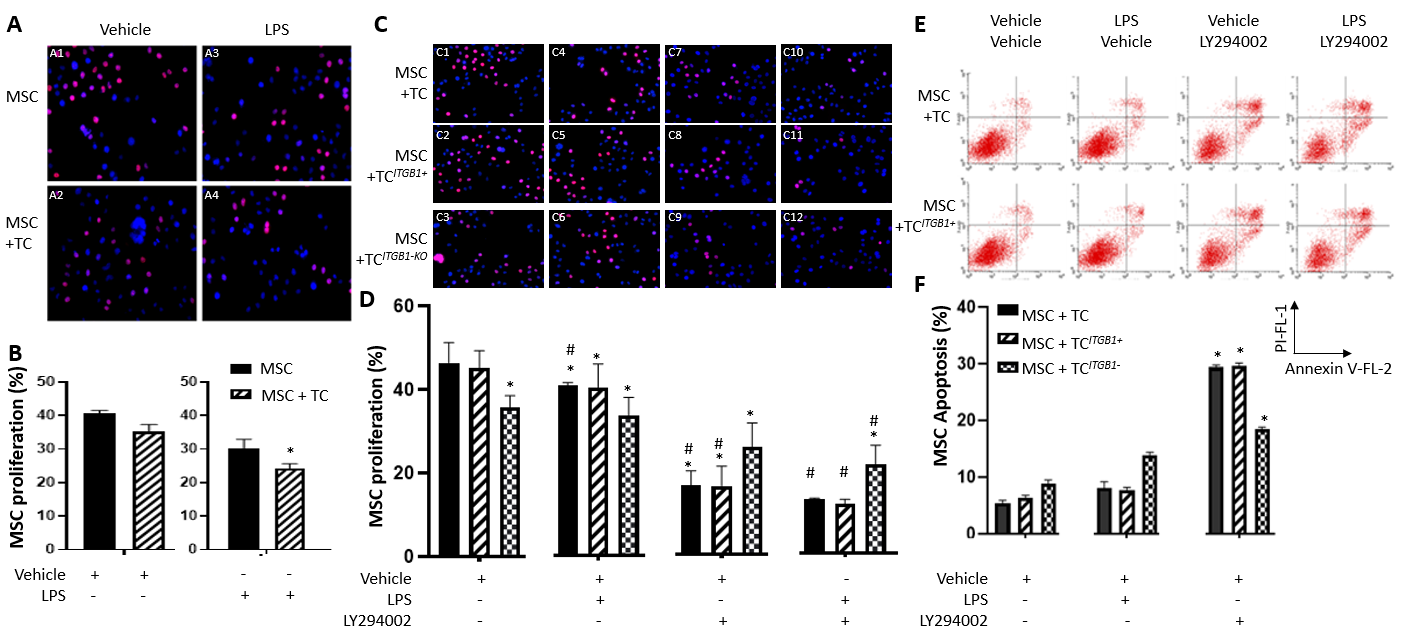

Supplement: Supplementary file 1 — Figure S1. [file JCMM-27-3980-s002.png]

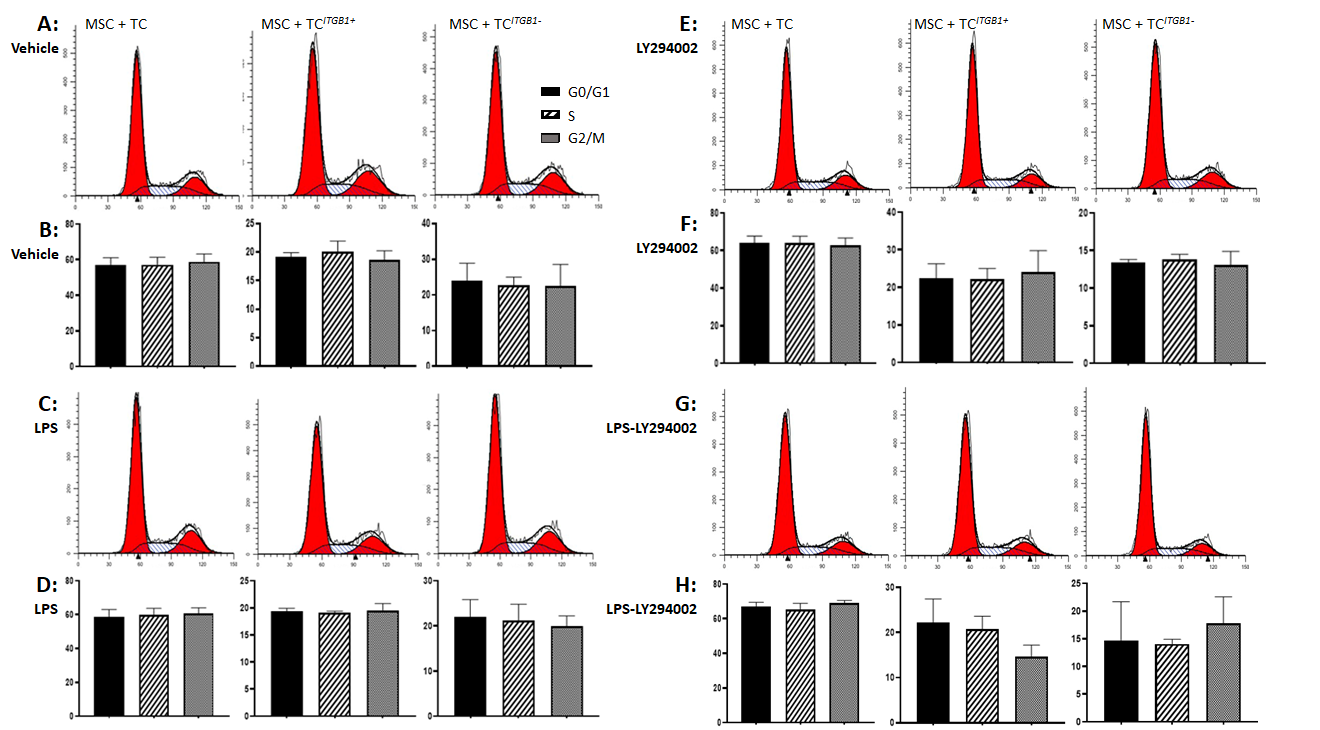

Supplement: Supplementary file 2 — Figure S2. [file JCMM-27-3980-s001.png]

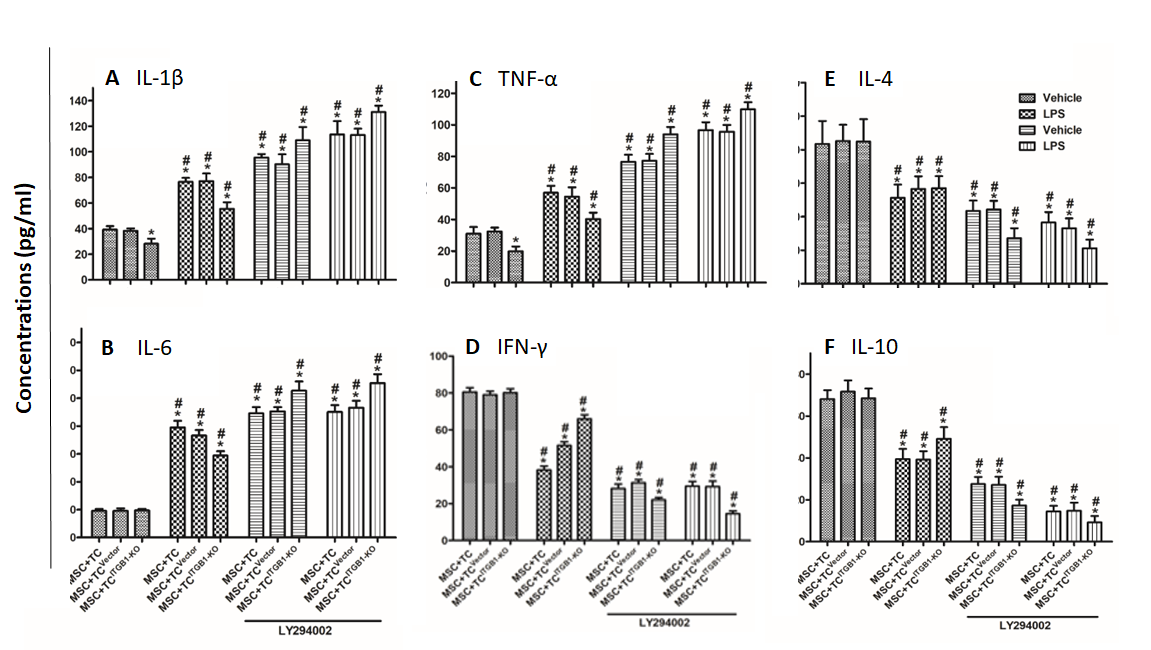

Supplement: Supplementary file 3 — Figure S3. [file JCMM-27-3980-s005.png]

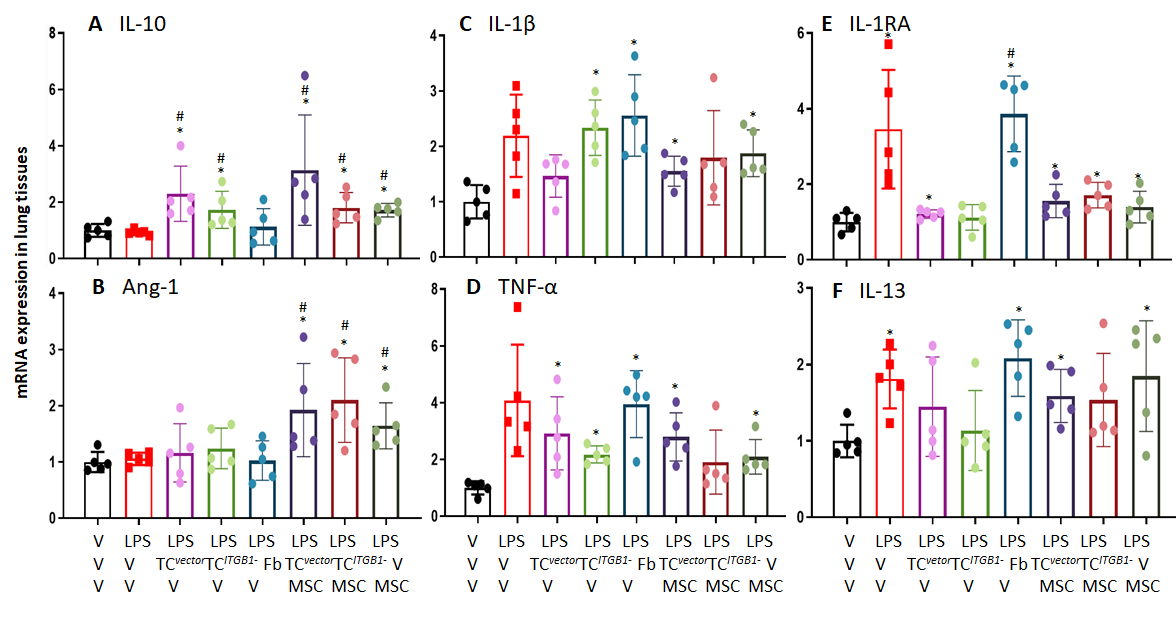

Supplement: Supplementary file 4 — Figure S4. [file JCMM-27-3980-s003.png]

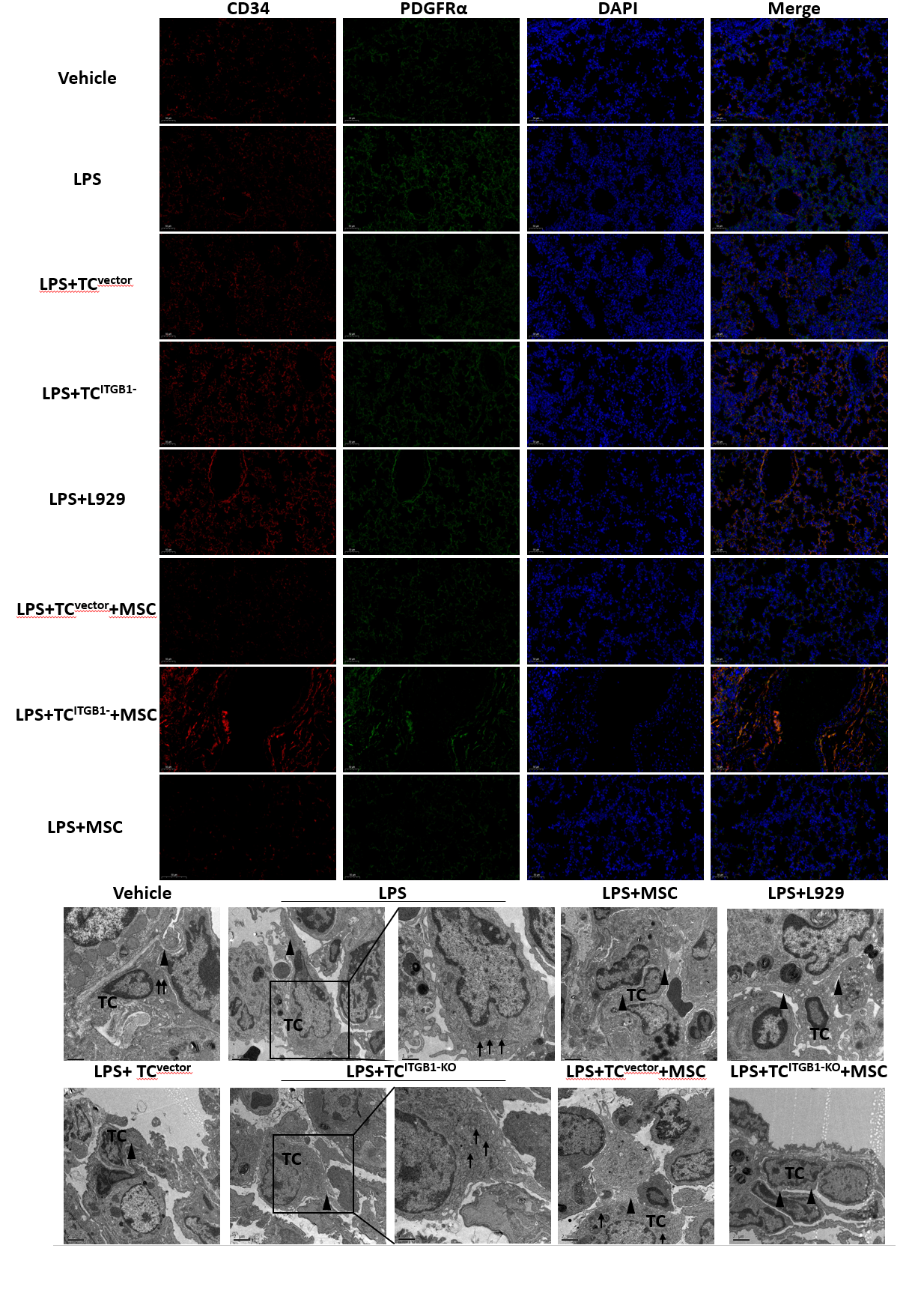

Supplement: Supplementary file 5 — Figure S5. [file JCMM-27-3980-s007.png]

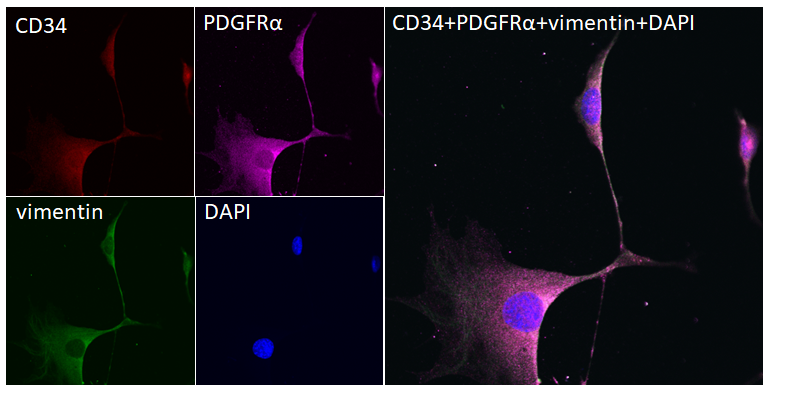

Supplement: Supplementary file 6 — Figure S6. [file JCMM-27-3980-s006.png]
