# Supplementary material for: Integrin beta1 mediates the effect of telocytes on mesenchymal stem cell proliferation and migration in the treatment of acute lung injury
Source: J Cell Mol Med. 2023 Oct 19;27(24):3980–94. doi: 10.1111/jcmm.17976 (PMC10746951; doi:10.1111/jcmm.17976)
Supplement: Supplementary file 7 — Table S1. [file JCMM-27-3980-s004.docx]

**Supplemental** **Table 1. Gene primer sequence used for quantitative RT-PCR.**

| **Gene names** | **Forward primer sequence** | **Reverse primer sequence** |
| --- | --- | --- |
| ***GAPDH***  ***IL-1β*** | AGATCATCAGCAATGCCTCCT  TCCGACCACCACTACAGCAAGG | TGAGTCCTTCCACGATACCAA  TGGGCAGGGAACCAGCATCTT |
| ***IL-4***  ***IL-6***  ***IL-10***  ***TNF-α***  ***IFN-γ*** | ACAGCAGTTCCACAGGCACAA  CCTTCGGTCCAGTTGCCTTCTC  ACTGCTCTGTTGCCTGGTCCTC  TGCTCCTCACCCACACCATCAG  AGAGTGTGGAGACCATCAAGGA | ACTCTGGTTGGCTTCCTTCACA  AGAGGTGAGTGGCTGTCTGTGT  GCCTTGATGTCTGGGTCTTGGTTC  TCCCAAAGTAGACCTGCCCAGAC  TGCGTTGGACATTCAAGTCAGT |

**Supplemental Table 2. Pathological scores**

| **Evaluation type** | **Degree** | **Area** | **Score** |
| --- | --- | --- | --- |
| **Hemorrhage (Hemorrhage volume)** | **Slight (+)** | **<20%** | **1** |
|  | **Moderate (++)** | **20%-50%** | **2** |
|  | **Severe (+++)** | **＞50%** | **3** |
| **Inflammation** | **Slight (+)** | **<20%** | **1** |
|  | **Moderate (++)** | **20%-50%** | **2** |
|  | **Severe (+++)** | **＞50%** | **3** |
| **Hyperplasia** | **Slight (+)** | **<30%** | **1** |
|  | **Moderate (++)** | **30%-50%** | **2** |
|  | **Severe (+++)** | **＞50%** | **3** |
